# Influence of the external breast prosthesis on the postural control of women who underwent mastectomy: Cross-sectional study

**DOI:** 10.3389/fonc.2022.920211

**Published:** 2022-08-29

**Authors:** Anna Koralewska, Małgorzata Domagalska-Szopa, Robert Łukowski, Andrzej Szopa

**Affiliations:** ^1^ Department of Developmental Age Physiotherapy, Medical University of Silesia, Katowice, Poland; ^2^ Medical Clinic Sanus, Zabrze, Poland; ^3^ Department of Physiotherapy, Medical University of Silesia, Katowice, Poland; ^4^ Neuromed, Rehabilitation and Medical Center, Katowice, Poland

**Keywords:** mastectomy, external breast prosthesis, postural control, stabilographic platform, posturography

## Abstract

Women after mastectomy may decide to either have a breast reconstruction or use an external breast prosthesis. Aim: The aim of the presented research was to evaluate the influence of external breast prosthesis on postural stability in women after mastectomy. Methods and Procedures: In the study 52 women after unilateral mastectomy took part. The study consisted of 4 parts: 1) anthropometric measurements; 2) measurements of upper limb circumference; 3) assessment of weight-bearing (WB); and 4) posturographic tests (PT). Outcomes and Results: Differences in the arm circumferences on the amputated (A) and non-amputated (NA) sides did not confirm the occurrence of lymphedema in limb on amputated side. The results of the WB between the A and NA body sides in both tested conditions, i.e., with open and closed eyes, showed no significant differences between the test with and without an external prosthesis. No statistically differences have been observed between posturometric parameters with and without breast prosthesis during both PT. In comparing the posturometric parameters between the PT with open and closed eyes, the sway path of the center of pressure was statistically significantly longer when eyes were closed in both conditions, i.e., with and without breast prosthesis. Conclusion and Implications: The finding show that 1) external breast prosthesis does not have a significant influence on the symmetry of loading on the A and NA body sides and on the postural stability of women after unilateral mastectomy and 2) exclusion visual control during PT increases postural instability in women after unilateral mastectomy.

## 1 Introduction

Breast cancer is considered the greatest oncological problem in developed countries and a constantly growing problem in developing countries (1–4). According to the National Cancer Registry, malignant neoplasms are the second most common cause of death in Poland, while breast cancer is the second most common cancer-related cause of death in women (15%) (5). Considering the predicted demographic changes in the Polish population and the fact that the highest percentage of cases is noted in individuals aged >50 years, it can be expected that by 2025, >80,000 women in Poland will develop breast cancer (6).

There are several well-established cancer treatment procedures such as radiotherapy, hormone therapy, chemotherapy, biological treatment, and surgical treatment.

The reduced invasiveness of breast cancer surgical treatment is a widely acknowledged preference; hence, there is a tendency to perform the breast-conserving procedure, which increase the quality of patient’s life (7, 8). Nevertheless, sometimes it is necessary surgical treatment to remove the entire tumor along with the entire mammary gland, the fascia of the pectoralis major, and the lymph nodes (Modified Radical Mastectomy or Simple Mastectomy), which is always associated with a structural and functional deficit (2, 9, 10).

A large proportion of women worldwide decide to undergo breast reconstruction after mastectomy. However, in Poland, only 20%–40% of women decide to undergo this type of surgery after breast removal. Women refuse breast reconstruction because of their age, fear of another surgery, postoperative complications, recurrence of cancer, or financial reasons. Due to the low percentage of breast reconstruction procedures, there is a need to use external breast prostheses in patients after mastectomy (11–13).

In studies on the population of women after mastectomy, the most attention is paid to issues related to psychologic parameters, such as lack of acceptance of one’s own body, reduced attractiveness, and sexuality (14–16). Some studies have also reported numerous functional disorders resulting mainly from an extensive wound, scarring, swelling, limited mobility in the joints of the shoulder girdle, and low muscle strength on the operated side (9, 13, 17).

Recently, post-mastectomy postural control disorders have become the subject of interest of many researchers (4, 9–11, 13, 18–27). The authors attempted to determine whether and how breast prosthesis affects body posture (28) and postural stability (11, 24). There is an agreement in both the existing literature and popular opinion that unilateral mastectomy results in postural control changes in women with breast cancer.

However, most of the abovementioned studies (4, 9–11, 13, 19, 25–27) assessed stability disorders in women after mastectomy by comparing them with their healthy counterparts. Considering the individual nature of regeneration of the body and primarily the course of compensation processes after treatment and unilateral breast amputation, it seems that the assessment of postural control disorders through inter-individual comparisons (women after mastectomy vs. healthy counterpart) may not be reliable.

Therefore, in this study, in addition to assessing the postural stability of women who underwent unilateral mastectomy, an attempt was made to evaluate the impact of external breast prosthesis (EBP) on postural stability by comparing the results of posturographic tests conducted on the same subject under two conditions—1) with EBP and 2) without EBP.

It has been hypothesized that postural stability disorders occur in women who undergo unilateral mastectomy and that the EBP plays a significant role in counteracting postural instability in this population. An additional aim of this study was to identify whether postural stability disorders in women who underwent unilateral mastectomy depended on the time since mastectomy was performed and time of using an EBP.

## 2 Materials and methods

A total of 52 women who underwent unilateral Modified Radical Mastectomy or Simple Mastectomy and who participated in the European Union program “You’re worth it” were analyzed. The study was conducted in cooperation with the Gliwice Oncology Center and the Amazon Clubs of Zabrze and Gliwice. The program focused on providing comprehensive care and rehabilitation to women who had breast cancer living in the Silesian region. The average age of the participants was 61.8 ± 10.8 years (range: 38–84 years). The mean body weight of the patients was 78.4 kg, body length was 160.0 cm, and body mass index was 30.7. All participants had undergone combination therapy (unilateral Modified Radical Mastectomy or Simple Mastectomy, chemotherapy, and/or radiotherapy). In the study group, 27 women underwent left-sided mastectomy and 25 women underwent right-sided mastectomy. The mean time from surgery was 6.5 ± 7.6 years. All study participants wore an EBP, which was selected by a skilled person. All study participants used a EBP at least during the day. All study participants met the inclusion and exclusion criteria. The characteristics of the study group are presented in **Table 1**.

**Table 1:** Characteristics of the participants.

Parameters	Mastectomy Group N=52		
	Mean ± SD	Median	Min–Max
Age (years)	61.8 ± 10.8	62.5	38 – 84
Height (cm)	160.0 ± 6.0	160.5	144 –173
Weight (kg)	78,4 ± 17.2	77.5	51–126
BMI (kg/m^2)^	30.7 ± 6.6	29.4	17.4 – 47.3
Time since surgery (years)	6.5 ± 7.6	3.0	0.5 – 35.0

The inclusion criteria were as follows: 1) female sex, 2) unilateral Modified Radical Mastectomy or Simple Mastectomy, 3) use of EBP for at least 12 h during the day, and 4) provision of a written informed consent to participate in the study.

The exclusion criteria were as follows: 1) dizziness, 2) imbalance or use of medications affecting the body’s balance, 3) nervous system diseases (Parkinson’s disease, post-stroke condition, peripheral nerves paralysis), 4) system disorders and skeletal disorders (posture defects, foot deformities), 5) rheumatic diseases, 6) condition after injuries, 7) metastases to the skeletal system, and 8) mental disorders (depression, dementia).

Before initiating the study, each participant was informed of the purpose and assumptions of the research project and the individual elements of the study. Participants were also informed that participation in the study was completely voluntary and that it was possible to withdraw from the research project without providing any reason. The study was conducted after obtaining written consent from participants. The research design received a positive opinion from the Bioethical Committee of the Medical University of Silesia in Katowice (Resolution No. KNW/0022/KB1/61/18). The study was conducted in accordance with the Declaration of Helsinki.

The examination consisted of four interrelated parts—1) anthropometric measurements; 2) measurements of the circumference of the upper limbs; 3) evaluation of the weight-bearing distribution; and 4) posturographic testing (center of pressure [CoP] measurements).

### 2.1 Anthropometric measurements

The height and weight of the test person were measured using a scale with a height gauge. The length of the lower limbs was measured using a tensile-resistant sewing tape, measuring the distance between the greater trochanter of the femur and medial ankle separately for the right and left lower limbs.

### 2.2 Measurements of upper limb circumference

Upper limb circumferences were measured using a tensile-resistant sewing tape. Measurements were conducted at two levels in each participant. The first measurement (brachial circumference) was made approximately 10 **cm** above the lateral epicondyle of the humerus, while the second measurement (forearm circumference) was made approximately 10 **cm** below this epicondyle.

### 2.3 Measurements of weight-bearing distribution and posturographic tests (CoP measurements)

During posturographic examinations, the participants stood barefoot in a relaxed standing position, with the upper limbs along the body and head facing forward. The distance between the medial ankles was approximately 3 cm.

Posturographic examination was performed under twoconditions: 1) with the EBP and then 2) without the prosthesis. Successively, in both conditions following measurements were made:

1. Weight-bearing distribution between the A and NA sides of the body with the eyes open and then with eyes closed
2. Posturographic test with eyes open and then with eyes closed

A force plate PDM, ZEBRIS (Germany) with FootPrint software, was applied for posturographic examination. Each measurement was recorded three times (3 trial, each lasted for 30 seconds with 30 sec pauses between trials). The mean values from three trials were used for future analysis.

CoP shifts and surface area of the CoP were the basis for the following posturometric parameters:

1) Path length of the CoP (SPL)
2) Lateral sway path of the CoP—the length of the short axis of the ellipse – the width of the ellipse (WoE)
3) Anterior–posterior sway path of the CoP—the length of the long axis of the ellipse – the height of the ellipse (HoE)
4) Area of the ellipse containing 95% of the recorded points of the projection of the center of pressure into the ground (AoE)
5) Angle of the ellipse determined by CoP (aoE)

**Figure 1:**
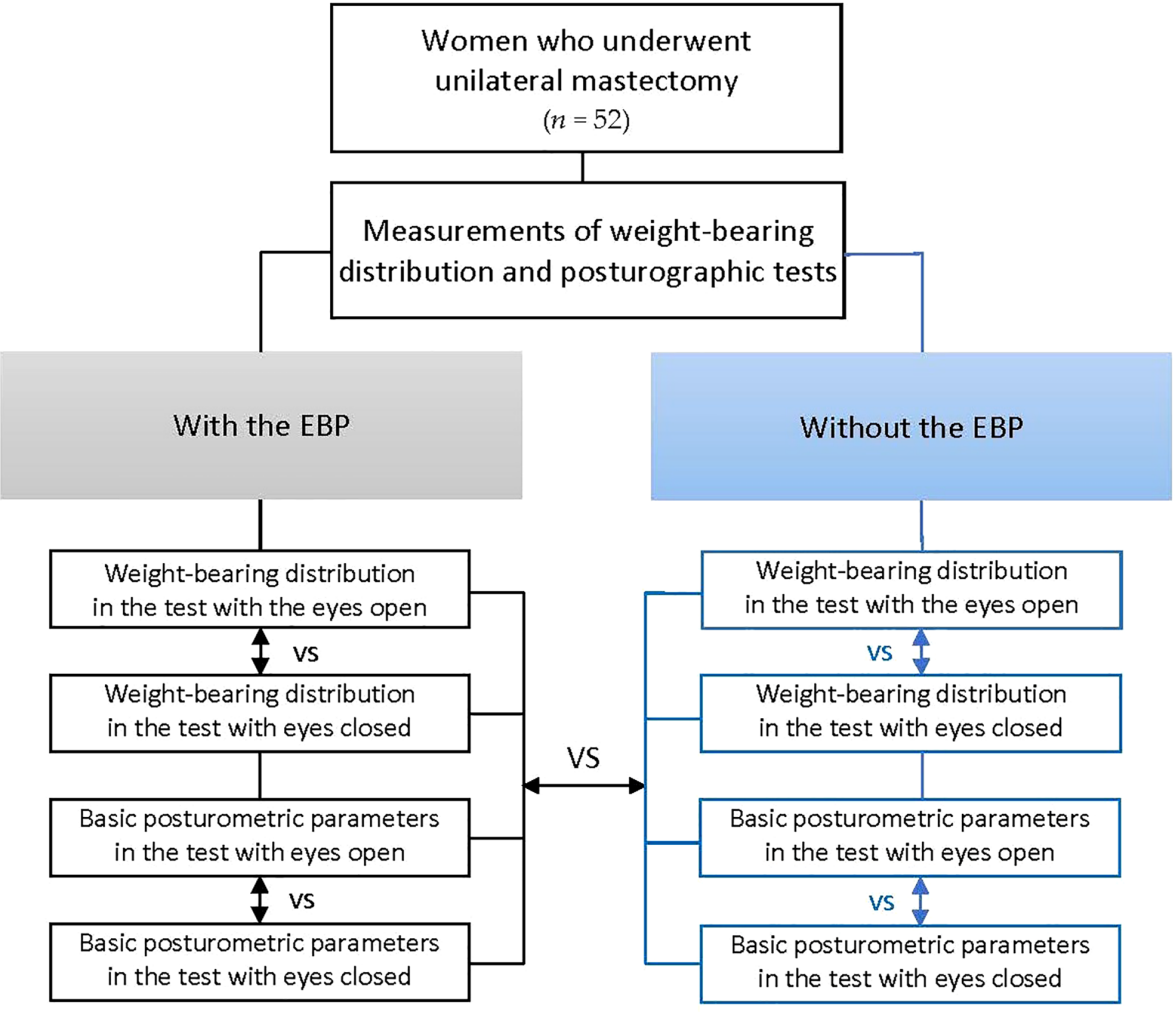
Flowchart describing the study flow. EBP, external breast prosthesis.

Flowchart describing the study flow is shown in **Figure 1**.

### 2.4 Statistical analysis

Statistical analyses were performed using IBM SPSS Statistics version 25. The data’s statistical distribution was identified using the Shapiro–Wilk test, and descriptive statistics were calculated. The differences in the weight-bearing distribution, between the A and NA sides of the body, were expressed by the absolute values of the percent load of the A and NA sides and the symmetry index (SI). SI was calculated using the following formula: │X_AM_-X_NA_│/avg (X_AM_, X_NA_) × 100%, where X_AM_ and X_NA_ are the values of a given parameter on both sides of the body – amputated (X_AM_) and non-amputated (X_NA_) (29). The differences in the parameters of the weight-bearing distribution between the A and NA sides of the body and posturometric parameters with and without the EBP, with the eyes open and closed, were analyzed using the Student’s t-test or Wilcoxon’s test. For the variables with a normal distribution, the Student’s t-test was used for statistical calculations, while for variables with a distribution other than the normal, the Wilcoxon test was used for statistical calculations. The statistical significance level was considered at a P-value <0.05. The Mann–Whitney U test was used to identify differences in upper limb circumferences between the A and NA sides. Spearman’s rank correlation test was used to calculate the correlation between the weight-bearing distribution and posturometric parameters and demographic data. Correlation coefficients were interpreted according to Altman’s recommendations: Rs<0.2, weak; 0.21–0.4, low; 0.41–0.6, moderate; 0.61–0.8, high; and 0.81–1, very high.

## 3 Results

Measurement of the circumference of the upper limbs aimed to identify the presence of lymphedema in the upper limb on the amputated side. The comparison of the corresponding circumferences (brachial and forearm circumferences) between the A and NA limbs are summarized in **Table 2**.

**Table 2:** Differences in upper limb circumferences at the brachial level (circumference 1) and at the forearm level (circumference 2) between the A and NA sides.

Parameters	A			NA			p-value
	Mean ± SD	Median	Min–Max	Mean ± SD	Median	Min–Max	
circumference 1- brachial	23.42 ± 3.86	22	18 – 33	22.06 ± 3.64	22	17 – 29	0.156
circumference 2- forearm	30.44 ± 4.54	30	21 – 42	28.71 ± 3.64	28	21 – 39	**0.039**

p-value – statistical significance test value; statistically significant differences are printed in bold; A, amputated side; NA, non-amputated side.

The comparative analysis did not show any statistically significant differences in arm circumferences between the A and NA sides. However, significant differences were found in forearm circumferences. The mean difference in the forearm circumferences did not exceed 2 cm.

Based on the value of the SI, participants were divided into the following three subgroups:

A) SI ≥20—the percentage of load is greater on the amputated side
B) SI ≥-20—the percentage of load is greater on the non-amputated side
C) - 20< SI <20—the percentage of load is equal or close to equal on the A and NA sides

The analysis of the distribution of the participants in terms of the weight-bearing distribution between the A and NA sides showed that the dominant tendency was to load both sides of the body evenly. Regardless of the test conditions (with a prosthesis, without a prosthesis, with eyes open and eyes closed), in more than half of the participants, the weight-bearing distribution was equal or close to equal (less than 5% of the difference) between the A and NA sides (**Table 3**).

**Table 3:** Characteristics of the symmetry index in individual trials.

Parameters	SI					
	eyes open			eyes closed		
	Mean ± SD	Median	Min–Max	Mean ± SD	Median	Min–Max
with EBP	-2.15 ± 17.33	-4	-48 – 48	-0.54 ± 16.84	2	-48 – 72
without EBP	0.38 ± 19.42	2	-56 – 56	1.54 ± 18.29	4	-56 – 48

EBP, external breast prosthesis; SI, symmetry index.

The results of the comparative analysis of the weight-bearing distribution between the A and NA sides of the body, expressed as the percentage of load on the A and NA side, and the SI in both tested conditions, i.e., with and without the EBP, as well as with eyes open and closed, showed no statistically significant differences between the test results performed with and without an external prosthesis (**Tables 4**, **5**).

**Table 4:** Comparison of the weight-bearing distribution between the A and NA sides during the trial with and without the EBP while maintaining a free-standing position with the eyes open and closed (N = 52).

Parameters	eyes open		eyes closed		eyes open	eyes closed
	with EBP	without EBP	with EBP	without EBP	p-value	p-value
	Mean ± SD	Mean ± SD	Mean ± SD	Mean ± SD		
load on the A (%)	49.46 ± 4.33	50.10 ± 4.86	49.87 ± 4.21	50.38 ± 4.57	0.327*	0.346*
load on the NA (%)	50.54 ± 4.33	49.90 ± 4.86	50.13 ± 4.21	49.62 ± 4.57	0.438	0.346*
SI	13.08 ± 11.43	15.31 ± 11.71	11.62 ± 12.10	13.54 ± 12.25	0.143	0.397

*Student’s t-test; p-value – statistical significance test value; EBP, external breast prosthesis; SI, symmetry index; A, amputated side; NA, non-amputated side.

**Table 5:** Comparison of the weight-bearing distribution between the A and NA sides during the test with eyes open and closed while standing alone with and without the EBP (N = 52).

Parameters	with EBP		without EBP		with EBP	without EBP
	eyes open	eyes closed	eyes open	eyes closed	p-value	p-value
	Mean ± SD	Mean ± SD	Mean ± SD	Mean ± SD		
load on the A (%)	49.46 ± 4.33	49.87 ± 4.21	50.10 ± 4.86	50.38 ± 4.57	0.394	0.662*
load on the NA (%)	50.54 ± 4.33	50.13 ± 4.21	49.90 ± 4.86	49.62 ± 4.57	0.394	0.662*
SI	13.08 ± 11.43	11.62 ± 12.10	15.31 ± 11.71	13.54 ± 12.25	0.328	0.185

*Student’s t-test; p-value, statistical significance test value; EBP, external breast prosthesis; SI, symmetry index; A, amputated side; NA, non-amputated side.

No statistically significant differences have been observed between the results of posturographic tests conducted with and without an EBP during tests with eyes open and closed (**Table 6**). The only significant difference was in the angle of the ellipse defined by the projection of the center of gravity. In the test with EBP, the angle of the ellipse showed negative values, while, in the test without prosthesis, it showed positive values. Negative values of the ellipse angle indicate that the resultant of the gravity forces runs diagonally toward the left. Conversely, positive values of this angle indicate that the resultant of gravity forces runs diagonally toward the right (**Table 6**).

**Table 6:** Comparison of the results of posturographic tests conducted with and without EBP during independent standing in a free standing position with eyes open and closed (N = 52).

Parameters	eyes open		eyes closed		open eyes	eyes closed
	with EBP	without EBP	with EBP	without EBP	p-value	p-value
	Mean ± SD	Mean ± SD	Mean ± SD	Mean ± SD		
SPL (cm)	67.70 ± 21.95	63.70 ± 14.74	73.78 ± 22.75	70.05 ± 18.54	0.260	0.116
WoE (cm)	2.15 ± 1.27	2.23 ± 1.91	2.01 ± 1.08	2.15 ± 2.14	0.572	0.920
HoE (cm)	4.07 ± 1.64	3.70 ± 1.43	4.39 ± 1.88	4.66 ± 1.87	0.227	0.141
AoE (cm²)	7.50 ± 6.85	6.62 ± 6.26	7.46 ± 6.00	8.87 ± 12.14	0.355	0.584
aoE (°)	-4.05 ± 17.37	5.37 ± 18.45	-3.40 ± 16.41	-1.59 ± 10.30	0.001*	0.756

*Student’s t-test; p-value, statistical significance test value; statistically significant differences are printed in bold; EBP, external breast prosthesis; SPL, path length of the CoP; WoE, width of the ellipse; HoE, height of the ellipse; AoE, area of the ellipse; aoE, the angle of the ellipse.

In comparing the posturometric parameters between the test conducted with eyes open and closed, in both conditions, i.e., with and without an EBP, statistically significant differences were noted in the path length of the CoP. The path of movement in the test conducted both with and without the EBP was statistically significantly longer in the test with eyes closed. Additionally, in the test conducted without the EBP, the HoE was significantly longer in the test with eyes closed. This indicates a significantly greater anterior–posterior CoP scavenging under conditions with visual inspection disabled (**Table 7**).

**Table 7:** Comparison of the results of the posturographic tests with eyes open and closed during self-standing in a free standing position during the tests with and without EBP (N = 52).

Parameters	with EBP		without EBP		with EBP	without EBP
	eyes open	eyes closed	eyes open	eyes closed	p-value	p-value
	Mean ± SD	Mean ± SD	Mean ± SD	Mean ± SD		
SPL (cm)	67.70 ± 21.95	73.78 ± 22.75	63.70 ± 14.74	70.05 ± 18.54	**0.001**	**0.000**
WoE (cm)	2.15 ± 1.27	2.01 ± 1.08	2.23 ± 1.91	2.15 ± 2.14	0.336	0.923
HoE (cm)	4.07 ± 1.64	4.39 ± 1.88	3.70 ± 1.43	4.66 ± 1.87	0.210	**0.004**
AoE (cm²)	7.50 ± 6.85	7.46 ± 6.00	6.62 ± 6.26	8.87 ± 12.14	0.707	0.059
aoE (°)	-4.05 ± 17.37	-3.40 ± 16.41	5.37 ± 18.45	-1.59 ± 10.30	0.728	0.078

p- value, statistical significance test value; statistically significant differences are printed in bold; EBP, external breast prosthesis; SPL, path length of the CoP; WoE, width of the ellipse; HoE, height of the ellipse; AoE, area of the ellipse; aoE, the angle of the ellipse.

In analyzing the correlation between the weight-bearing distribution with demographic data (e.g., age of the participants, time since surgery, height, BMI, and body weight), no significant correlations were found. Exploration the relationship between posturometric parameters and demographic data revealed a several significant weak correlations. The following posturometric parameters: SPL, WoE, HoE and AoE were positively related to age of participants, which means, that the older participant, the worse the posturometric parameters (**Table 8**).

**Table 8:** Correlation of posturometric parameters with the age of the participants.

Posturometric parameters	Age of the participants							
	with EBP				without EBP			
	eyes open		eyes closed		eyes open		eyes closed	
	r	p-value	r	p-value	r	p-value	r	p-value
SPL (cm)	**0.31**	**0.025**	**0.40**	**0.003**	0.26	0.066	0.31	0.028
WoE (cm)	**0.30**	**0.033**	0.20	0.148	0.27	0.052	0.19	0.180
HoE (cm)	**0.31**	**0.025**	0.25	0.075	0.18	0.207	0.12	0.406
AoE (cm²)	**0.37**	**0.007**	**0.30**	**0.033**	**0.32**	**0.019**	0.20	0.162
aoE (°)	-0.01	0.931	-0.02	0.883	0.15	0.288	0.06	0.693

r, Spearman’s rank correlation coefficient; p-value, value of the correlation significance test; statistically significant differences are printed in bold; EBP, external breast prosthesis; SPL, path length of the CoP; WoE, width of the ellipse; HoE, height of the ellipse; AoE, area of the ellipse; aoE, the angle of the ellipse.

## 4 Discussion

This study aimed to assess the impact of EBP on postural stability of women who underwent unilateral mastectomy by comparing the results of the posturographic tests conducted on the same patient under two conditions, i.e., with and without EBP. Although the initial hypothesis that the EBP plays a significant role in maintaining a stable standing posture in women who underwent unilateral mastectomy seemed obvious and significant differences were expected, the study suggests the opposite. Not only did the conducted statistical analysis not confirm the differences in the weight-bearing distribution between the A and NA sides of the body in natural conditions, i.e., with EBP (as already presented above), there were also no significant differences between the tests conducted with and without the EBP. Moreover, in comparing the measurements of postural stability while maintaining a standing position with and without the EBP, no significant differences in the basic posturometric parameters were found, neither in the tests with open eyes nor in the tests with eyes closed.

Additionally, the differences in the arm circumferences on the A and NA sides did not confirm the occurrence of lymphedemas typical for the amputated limb after mastectomy. The hypothesis that the EBP provides better postural stability in women after unilateral mastectomy – in the context of the obtained results – was not confirmed by the conducted studies.

The question whether EBP influences the postural stability of women who underwent mastectomy was not answered by the previous studies. Moreover, the literature on the subject indicates that the EBP does not play such a significant role as it could be assumed in the postural control of women who underwent unilateral mastectomy (11, 28, 30). Karczewska et al., based on studies assessing the influence of external prostheses on the dynamic balance of women who underwent unilateral mastectomy, found that the breast prosthesis did not affect the quality of equivalent reactions in the dynamic study (24). Similarly, in the extensive study conducted by Manikowska et al., who assessed the impact of external breast prostheses of three different weights on the measures of stability in women who underwent unilateral mastectomy, no statistically significant differences were found between posturographic tests conducted with different prostheses and without a breast prosthesis. It was interesting that the values of the weight-bearing distribution and posturometric parameters with the prosthesis, the mass of which was 50% of the amputated breast mass, were closest to the results obtained in the control group consisting of healthy counterparts (11). While examining the influence of EBP of different weights on the activity of the extensor muscles of the spine on the A and NA sides, Hojan et al. also found that the weight of the EBP did not affect the body posture of these women (28). In their subsequent studies, they presented scientific evidence that the weight of the EBP did not affect the biomechanics of the torso (30). Both the previous and presented results may indicate the activity of compensatory mechanisms activated as a result of the breast amputation procedure and organism’s efforts to compensate for the postural symmetry disorder and its control.

Another goal of this study was to assess the dynamic postural stability of women who underwent unilateral mastectomy, a comparison of the stability measures in the posturographic test with eyes open and test excluding visual control in a standing position. Comparison of the postural stability measurements while maintaining the standing position with the EBP in place (i.e., under natural conditions) revealed significant differences between their values recorded during the test under visual control conditions (test with eyes open) and its switch-off conditions (test with eyes closed). The basic posturometric parameter, i.e., SPL, was almost 10% longer when the visual control was turned off than that with the eyes open. Therefore, the results of this study may indicate impairment of dynamic postural control, i.e., under conditions of disabled visual control in women who underwent unilateral mastectomy. Moreover, the abovementioned findings are confirmed by the results of previous scientific studies. Głowacka-Mrotek et al., in studies conducted on a group of patients who underwent unilateral mastectomy, comparing them to a group of healthy women, noted statistically significantly worse results in terms of stability measurements for both open and closed eyes in the group of women who underwent mastectomy. In the posturographic test with eyes open, this concerned the following parameters: maximum back deviation, maximum forward deviation, average Y deviation, average Y velocity, path length, and path surface area (13). However, in the sample with the exception of visual inspection, it concerned maximum backward deviation, maximum forward deviation, mean Y deviation, and path length. Moreover, these results confirm our observations regarding significantly higher HoE (which corresponds to the maximum backward and forward deviation range) recorded in the test without EBP with eyes closed. More recently, Mangone et al. compared the dynamic stability of women who underwent unilateral breast amputation to that of their healthy counterparts. They found that the stability measurements obtained from women who underwent mastectomy are worse both under visual control and after its switching off. They noted significantly worse results in both length of the ellipse and area of the ellipse plotted by the CoP in the test with eyes closed (10). In turn, Montezuma et al. recorded significantly higher maximum CoP velocity in both the tests with eyes open and eyes closed in comparative studies of women who underwent mastectomy and a control group of healthy women. Additionally, these results were significantly worse when the visual inspection was turned off in both groups (4). The abovementioned observations were also confirmed in the literature review on balance and gait studies in women who underwent mastectomy compared to their healthy counterparts (26). Despite the fact that this project did not involve comparative studies with healthy counterparts, the abovementioned previous studies lead to the conclusion that the posturometric parameters obtained in this study differ from the results of the population of healthy women presented in other studies and indicate the presence of symptoms of postural instability in the studied population. In both the previous studies and our study, the stability measurements were correlated with the age of the participants. Most posturometric parameters were worse in older participants. Deterioration of postural stabilization is an indispensable element of the aging process and a physiological phenomenon (31).

It can be assumed that the postural control systems in patients who underwent unilateral mastectomy rely largely on the visual feedback needed to maintain an upright body posture. Therefore, disabling visual control may expose the imperfections of postural control based on proprioceptive mechanisms in women after mastectomy. Some scientific reports that assessed the effect of treatment adjunct to mastectomy, such as neurotoxic chemotherapy, indicate that they may induce symptoms of peripheral neuropathy and lead to disturbances or even loss of proprioception (32–35).

While previous studies referred to the stability measurements obtained in tests with EBP (e.g., of different weights), often with results obtained from healthy counterparts (independent sample comparisons), which could raise doubts regarding homogeneity of the studied groups, our study compared dependent samples, i.e., stability measurements obtained in the tests with the target breast prosthesis with which the patient functions on a daily basis and also without it.

Additionally, the tests conducted with and without the target EBP were compared under two conditions—with and without visual inspection. To the best of our knowledge, these are the first studies using such a methodology.

The study results show that 1) postural stability disorders occur in women who underwent unilateral mastectomy after switching off visual control and 2) EBP does not have a significant influence on the symmetry of loading on the A and NA sides of the body and on the postural stability of women after unilateral mastectomy.

Based on the obtained results, the following conclusion can be drawn: the deficiencies of postural control in women after mastectomy indicate the need to include proprioceptive training as an element of rehabilitation of women after mastectomy.

## 5 Limitations

Our study has some limitations. Although in the case of the strictly defined purpose of the presented study, it did not matter that much, the study population varied considerably in terms of age, time since mastectomy, and adjuvant treatment methods. Therefore, in further stages of the study, the abovementioned limitations will be considered.

However, the most important limitation was the not fully recognized condition of lymphedema in the upper limb on the amputation side. It is necessary to consider the natural differences between the weight of the upper limbs, e.g., dominant and non-dominant, and this has not been considered in the presented studies. Therefore, in follow-up studies, a segmental analysis of body mass composition, especially the percentage of water in the upper limbs, should be included.

## Data availability statement

The original contributions presented in the study are included in the article/supplementary material. Further inquiries can be directed to the corresponding author.

## Ethics statement

The studies involving human participants were reviewed and approved by Bioethical Committee of the Medical University of Silesia in Katowice (Resolution No. KNW/0022/KB1/61/18). The patients/participants provided their written informed consent to participate in this study.

## Author contributions

AK and MD-S: Conceptualization and writing – original draft. AK and MD-S: Investigation. AK and RŁ: Data curation. AS: Visualization. All authors contributed to the article and approved the submitted version.

## Acknowledgments

We would like to thank all the women who took part in the study and the team of Medical Clinic “Sanus” in Zabrze.

## Conflict of interest

The authors declare that the research was conducted in the absence of any commercial or financial relationships that could be construed as a potential conflict of interest.

## Publisher’s note

All claims expressed in this article are solely those of the authors and do not necessarily represent those of their affiliated organizations, or those of the publisher, the editors and the reviewers. Any product that may be evaluated in this article, or claim that may be made by its manufacturer, is not guaranteed or endorsed by the publisher.
